# Conductive Ink-Coated Paper-Based Supersandwich DNA Biosensor for Ultrasensitive Detection of *Neisseria gonorrhoeae*

**DOI:** 10.3390/bios13040486

**Published:** 2023-04-18

**Authors:** Niharika Gupta, D. Kumar, Asmita Das, Seema Sood, Bansi D. Malhotra

**Affiliations:** 1 Department of Biotechnology, Delhi Technological University, Delhi 110042, India; 2 Department of Applied Chemistry, Delhi Technological University, Delhi 110042, India; 3 Department of Microbiology, All India Institute of Medical Sciences, Ansari Nagar, New Delhi 110016, India

**Keywords:** DNA biosensor, *Neisseria gonorrhoeae*, conductive ink, electrochemical, infectious diseases

## Abstract

Herein, we report results of the studies relating to the development of an impedimetric, magnetic bead-assisted supersandwich DNA hybridization assay for ultrasensitive detection of *Neisseria gonorrhoeae*, the causative agent of a sexually transmitted infection (STI), gonorrhea. First, a conductive ink was formulated by homogenously dispersing carboxylated multiwalled carbon nanotubes (cMWCNTs) in a stable emulsion of terpineol and an aqueous suspension of carboxymethyl cellulose (CMC). The ink, labeled *C5*, was coated onto paper substrates to fabricate *C5*@paper conductive electrodes. Thereafter, a magnetic bead (MB)-assisted supersandwich DNA hybridization assay was optimized against the *porA* pseudogene of *N. gonorrhoeae*. For this purpose, a pair of specific 5′ aminated capture probes (SCP) and supersandwich detector probes (SDP) was designed, which allowed the enrichment of target gonorrheal DNA sequence from a milieu of substances. The SD probe was designed such that instead of 1:1 binding, it allowed the binding of more than one T strand, leading to a ‘ladder-like’ DNA supersandwich structure. The MB-assisted supersandwich assay was integrated into the *C5*@paper electrodes for electrochemical analysis. The *C5*@paper electrodes were found to be highly conductive by a four-probe conductivity method (maximum conductivity of 10.1 S·cm^−1^). Further, the biosensing assay displayed a wide linear range of 100 aM-100 nM (10^9^ orders of magnitude) with an excellent sensitivity of 22.6 kΩ·(log[concentration])^−1^. The clinical applicability of the biosensing assay was assessed by detecting genomic DNA extracted from *N. gonorrhoeae* in the presence of DNA from different non-gonorrheal bacterial species. In conclusion, this study demonstrates a highly sensitive, cost-effective, and label-free paper-based device for STI diagnostics. The ink formulation prepared for the study was found to be highly thixotropic, which indicates that the paper electrodes can be screen-printed in a reproducible and scalable manner.

## 1. Introduction

Sexually transmitted infections (STIs) remain one of the major medical problems, with more than 1M infections being acquired every day worldwide [1]. Gonorrhea, caused by the bacteria *Neisseria gonorrhoeae* (NG), is a curable STI having a very high incidence rate. The World Health Organization (WHO) has considered it as one of three STIs of global concern and has estimated eighty-two million new cases of gonorrhea in 2020 worldwide [2]. Factors that contribute to the high prevalence of gonorrhea include lack of access to testing and treatment, inadequate prevention efforts, and stigma surrounding STIs. Untreated gonorrhea can lead to serious complications, including pelvic inflammatory disease, infertility, neonatal blindness, ectopic pregnancy, and an increased risk of HIV transmission [1,3,4]. Another crucial factor to consider is the alarmingly increasing cases of antimicrobial-resistant (AMR) gonorrhea worldwide [2]. Thus, timely diagnosis and proper intervention is required to decrease the incidence of this STI.

The conventional detection strategies for *Neisseria gonorrhoeae* (NG) presently include culture techniques, microscopy, and the polymerase chain reaction (PCR). These techniques, though sensitive, are expensive, time-consuming, laborious, and cannot be performed outside of the laboratory [1,5]. Biosensors can prove to be essential for improving access to timely and accurate diagnosis, particularly in resource-limited settings where traditional laboratory-based testing may not be readily available. They can allow for the rapid detection of infections including gonorrhea at the point of care (POC), often providing results within minutes, which may help to expedite the treatment and reduce the risk of transmission [3,6,7]. In addition, biosensors can reduce the need for expensive and time-consuming laboratory testing, making them an attractive option for both healthcare providers and patients. Different kinds of DNA biosensors have been developed in recent years for the detection of various pathogens, such as *Vibrio cholerae* [8], *Haemophilus influenzae* [9], SARS-CoV-2 [10,11], Zika virus [12,13], etc. The high fidelity of DNA base pairing allows for the design and development of different kinds of DNA-based sensing mechanisms [3,6,10].

A few attempts to detect gonorrheal DNA have been made in the recent past. These studies have mostly focused on the development of amplification-based approaches for the visual detection of NG [3,6]. We have also previously reported the electrochemical detection of PCR amplicons of NG [14]. Visual detection is often semi-quantitative and amplification-based approaches suffer from limitations similar to those of other nucleic acid amplification assays (NAATs), e.g., the use of expensive reagents and the requirement of specific conditions (high temperature, etc.). In this context, supersandwich DNA hybridization is an attractive technique that alleviates the limitations associated with amplification-based assays and provides exceptionally high sensitivity as compared to traditional hybridization-based biosensors [15,16,17]. This method provides an enzyme-free signal amplification, can be operated at room temperature, and can be integrated into different biosensing modalities [15,16,18]. Supersandwich amplification-based biosensors have been applied for many applications including pathogen detection [19], and miRNA detection [16] among others. 

The paper-based biosensors can prove to be advantageous for screening, monitoring, and detection of gonorrhea, as they are cost-effective, can be miniaturized, and can be used in low-resource settings. Different kinds of paper-based analytical devices have been reported in literature for the detection of pathogenic DNA [9,20,21,22]. Electrochemical paper-based devices have gained interest owing to their high accuracy, quantitative nature, ease of use, and applicability at the PoC [23,24]. Nanomaterial-enhanced, paper-based screen-printed electrodes (SPEs) have recently gained popularity as a low-cost and sensitive option to traditional diagnostic methodologies [23,25,26]. With the advent of papertronics, new-age paper-based devices have emerged that can be utilized for various electrochemical applications including biosensors. The development of conductive inks has further driven the growth of paper-based devices and vice versa. Conductive inks are materials that can be printed or applied in a thin layer to a substrate and can be used to create electronic circuits and devices. Conductive inks have emerged as low-cost options for the fabrication of paper-based biosensors [27,28]. These inks can be utilized for the fabrication of biosensors towards the detection of several different analytes, including DNA, hormones, metal ions, etc. [27,28].

In this work, we have fabricated a conductive ink-coated, paper-based impedimetric DNA supersandwich hybridization assay for highly sensitive and label-free detection of *N. gonorrhoeae*. For the conductive ink (named *C5*), multiwalled carbon nanotubes (MWCNTs) have been used as the conductive filler. MWCNTs possess a high aspect ratio and high surface area, making them highly conductive and suitable for many applications. In addition, MWCNTs have good adhesion to various substrates, including plastics and paper, which makes them well-suited for use in flexible and lightweight devices. MWCNTs have good mechanical strength and can withstand high temperatures, which makes them suitable for use in different electrochemical applications. The *C5* ink was chosen from a series of inks for its optimum rheological behavior and coated onto paper substrates manually (*C5*@paper). Further, a magnetic bead (MB)-assisted DNA supersandwich assay was optimized against the *porA* pseudogene sequence of NG. The use of magnetic beads offers several advantages, including simplification of the sample enrichment process, the ability to be used in high-throughput assays, and automation. They can separate a target analyte from a milieu of substances owing to magnetic separation and greatly ease the purification process. The MB-assisted supersandwich assay was integrated with the *C5*@paper electrode for label-free detection of NG using electrochemical impedance spectroscopy (EIS). 

## 2. Materials and Methods

### 2.1. Materials and Instrumentation

Carboxylated MWCNT (cMWCNT) and polysorbate 80 (PS80) were procured from SRL Pvt. Ltd. (Mumbai, India). Carboxymethyl cellulose (CMC; high viscosity) and terpineol were purchased from Central Drug House (P) Ltd. (CDH, New Delhi, India). The oligonucleotide probes were procured from IDT (Coralville, IA, USA) (Table 1). Carboxylated magnetic beads (MBs; Dynabeads Carboxylic Acid^TM^ MyOne^TM^) were purchased from Thermo Fisher (Waltham, MA, USA). Freshly prepared phosphate-buffered saline (PBS, 0.1 M) of pH 7 supplemented with 5 mM ferro-ferricyanide as redox probes was utilized in the electrochemical studies, unless otherwise specified. All experiments were conducted in triplicates (n = 3) unless otherwise specified.

The ink formulations were analyzed for viscosity and thixotropy on an Anton Paar rheometer by the parallel plate (PP40, diameter 40 mm) method. All rheology experiments were conducted at 25 °C. The *C5*@paper electrodes (1 × 1 cm^2^) were characterized for surface morphology via different techniques, including scanning electron microscopy (SEM), field emission SEM (FESEM), and atomic force microscopy (AFM). For SEM and FESEM analysis, the samples were dried under an infrared lamp for 30 min to eliminate any adsorbed moisture and sputter coated with Au for electrical conductivity. SEM was conducted on a ZEISS EVO 18 instrument at 10 kV acceleration voltage. FESEM was performed on a JEOL JSM-7800F Prime instrument. AFM studies were conducted on an Asylum Research MFP3D-BIO instrument in intermittent contact (tapping) mode. The conductivity of the *C5*@paper electrodes was analyzed via a four-probe conductivity meter (SES Instruments, India) (probe distance = 2 mm) at room temperature (25 °C). Cyclic voltammetry (CV) and EIS studies were performed on a single-channel Autolab potentiostat/galvanostat (Metrohm AG, Herisau, Switzerland). All CV studies were conducted at scan rate of 50 mV·s^−1^ and EIS studies were conducted at 0.01 V within the frequency range of 0.1–10^5^ Hz.

### 2.2. Design of Oligonucleotide Probes

The supersandwich oligonucleotide probes were designed using NCBI-Primer BLAST, NCBI Nucleotide BLAST, and Clustal Omega. Briefly, a library of different sets of primers was prepared using NCBI-Primer BLAST, and their specificity was determined by aligning them with gonorrheal and non-gonorrheal genomic DNA sequences using NCBI Nucleotide BLAST. The non-gonorrheal DNA sequences used for alignment included those from *N. meningitidis*, commensal *Neisseria* species (*N. sicca*, *N. lactamica*, *N. mucosa*), *Chlamydia trachomatis*, *Ureaplasma urealyticum*, *Enterococcus faecalis*, *Escherichia coli*, *Staphylococcus aureus*, *Klebsiella pneumoniae*, *Mycoplasma genitalium*, and *Lactobacillus fermentum*. The forward primer from the set of probes having 100% similarity with gonorrheal DNA and having no or little similarity with non-gonorrheal DNA was selected as the capture probe (SCP), and an adjacent specific sequence was selected as the conventional detector probe (DP). For the supersandwich assay, the detector probe (SDP) sequence consisted of the DP sequence ligated to the SCP sequence in 5′-3′ direction. The details of all the probes utilized in the study are given in Table 1. The alignment of the probes with the target DNA sequence is given in Section S1 of the Supplementary Information. Gel electrophoresis studies were conducted to determine the probe efficiency in forming a supersandwich duplex with the target DNA sequence.

### 2.3. Formulation of the Conductive Ink

For ink formulation, different amounts of CMC (1, 2.5, and 5% *w*/*v*) were dissolved in deionized (DI) water. These CMC dispersions were then mixed with terpineol (2:1 ratio) separately. Polysorbate 80 (PS80) was used as the stabilizer, as terpineol has limited solubility in water and CMC does not interact with organic solvents. The resulting emulsions, designated as CMC_1_, CMC_2.5,_ and CMC_5_, respectively, were stirred vigorously on a magnetic stirrer (~1000 rpm, 2 h). CMC_5_ was chosen as the base for the conductive ink after rheological assessment. cMWCNTs were added to CMC_5_ (cMWCNT:CMC ratio of 1:1) with vigorous stirring (~1000 rpm, overnight). The resulting conductive emulsion was probe ultrasonicated in an ice bath at a pulse of 1 s and 70% amplitude for 20 min, with pauses at every 5 min to prevent overheating. The resulting conductive ink, designated as *C5*, was stored in a closed container at low temperature (4 °C–10 °C) to prevent evaporation of the solvent. 

### 2.4. Rheological Studies

The fluid properties of ink are imperative in understanding its behavior during the coating/printing process. The emulsion bases (CMC_1_, CMC_2.5,_ and C_5_) for the conductive ink, prepared with different amounts of CMC, were assessed for rheology by a plate-plate rheometer at 25 °C. The change in the shear viscosity of the emulsion bases was analyzed within the shear rate range of 0.1–100 s^−1^. Further, a 3-interval thixotropy test (3iTT) was utilized for analyzing the recovery of the viscosities of the 3 emulsion bases after stress removal. This test can be used to simulate screen printing and other coating processes, wherein an abrupt increase in stress is observed during the printing/coating process. The emulsion bases were pre-sheared at 0.1 s^−1^ for 20 s before the 3iTT. Thereafter, three intervals of different shear rates were applied: 0.1 s^−1^ for 30 s (first interval), 200 s^−1^ for 15 s (second interval; simulation of the printing process), and recovery at 0.1 s^−1^ for 120 s. 

### 2.5. Fabrication of C5@paper Electrodes

The *C5*-coated paper electrodes (*C5*@paper) were fabricated by first coating drawing paper (200–300 gsm, 6 × 6 cm^2^) with the *C5* ink using a commercially available paint brush (Faber-Castell). This *C5*-coated paper was then cut into strips having dimensions of 2 × 2 cm^2^, which were then annealed at 120 °C for 10 min to allow evaporation of the solvent (terpineol and water). The effect of the number of coats (*n*) on the conductivity of the *C5*-coated paper was assessed by measuring the conductivity after each coat with the 4-probe conductivity meter. For conductivity studies, the *C5*-coated paper strips were cut into pieces of size 1 × 1 cm^2^ each. The conductivity values of 4 strips (1 × 1 cm^2^; 12 readings in total) were recorded at 25 °C and their mean value was reported for each coat. For electrochemical studies, the strips were cut into strips with dimensions of 2 × 1 cm^2^ to fabricate the *C5*@paper electrodes. All electrodes were stored in dark, dry conditions until further use.

### 2.6. Electrochemical Magnetic DNA Supersandwich Assay

**Scheme 1:**
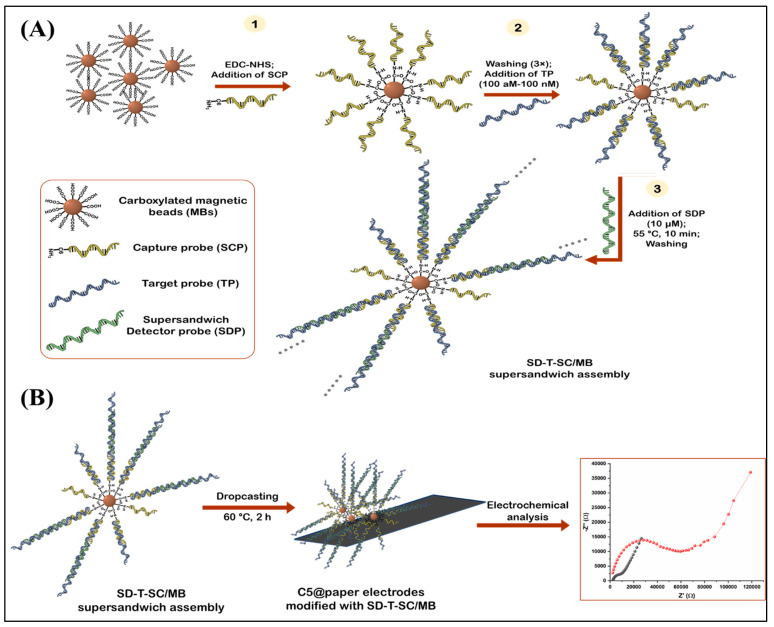
Schematic diagram depicting (**A**) the MB-assisted supersandwich hybridization assay for *N. gonorrhoeae* detection, and (**B**) subsequent integration with the *C5*@paper electrodes for electrochemical analysis via EIS.

The detailed protocol for the immobilization of SCP onto the MBs is provided in Section S2 of the Supplementary Information. Scheme 1 depicts the various steps involved in the electrochemical, MB-assisted biosensing assay.

#### 2.6.1. Supersandwich Assay

Five milligrams of SCP/MBs were washed with DI water (3×) and dispersed in 500 µL of DI water, making the final concentration 10 mg·mL^−1^. Aliquots of 50 µL were made in different test tubes and 50 µL of TP was added to each tube in the concentration range of 100 aM–100 nM (5 zmol–5 pmol). Thereafter, 50 µL of SDP (10 µM) was added to each reaction tube and incubated at 55 °C for 10 min with mixing. This SD-T-SC/MB supersandwich assembly was washed with DI water (3×) to remove unbound probes, and redispersed in 30 µL PBS buffer for electrochemical analysis.

#### 2.6.2. Electrochemical Analysis

For the electrochemical studies, the SD-T-SC/MB assembly dispersed in 30 µL of PBS was drop cast onto the *C5*@paper electrodes. These modified electrodes were slowly dried at 60 °C for 2 h. Thereafter, impedance measurements for every electrode were recorded in PBS (pH 7) supplemented with 5 mM ferro-ferricyanide. The measurements were made in the range of 0.1–10^5^ Hz at 0.01 V. A calibration curve was plotted between the net change in charge transfer resistance (R_ct_) of the *C5*@paper electrodes in the presence of different concentrations of TP (as compared to negative control) and the concentration of TP.

### 2.7. Real Sample Analysis

For practical applications, it is imperative that the electrochemical biosensing assay works in clinical settings and can specifically detect gonorrheal DNA from among a mixture of different substances. To assess this, the biosensing assay was first tested against gonorrheal genomic DNA. For this, DNA from *N. gonorrhoeae* bacterial culture was extracted using the boiling method. Briefly, a few colonies from the culture plate were dispersed in autoclaved, double distilled water (1 mL) and incubated at 90 °C for 1 h for inducing cell lysis. Then, the heat-lysed cell suspension was taken and centrifuged at 1200 rpm for 5 min to separate cell debris. The pellet was discarded and the remaining supernatant was stored at −20 °C until further use. The DNA was quantified using nanospectrophotometer (Nanodrop). It should be noted that DNA extracted using this method is often contaminated with RNA and other cellular components such as proteins. Hence, the purity level of such samples is often lower than the ideal values. This method was chosen for DNA extraction as it is quicker than the buffer-based commercial methods, requires fewer reagents, and is widely used in clinical labs for isolating DNA from cervical/urethral swabs—the samples utilized in gonorrhea testing.

Different dilutions of the extracted DNA were prepared, which were then sheared into smaller fragments by bath ultrasonication for 10 min, and denatured by incubation at 95 °C for 5 min. This ensured the denaturation of any residual, contaminating RNA and proteins in the samples. Thereafter, the protocol mentioned in the previous section was repeated to determine the concentration of gonorrheal DNA. 

As clinical samples (particularly from female subjects) are known to contain DNA from different bacterial species, the specificity of the biosensing assay was further assessed by testing it against a mixture of gonococcal DNA with non-gonococcal DNA extracted from different bacteria. The DNA samples were extracted by the boiling method discussed above; hence, they also contained interfering RNA and proteins. The details of the bacterial species used in the study are given in Section S3. The response of the electrochemical biosensing assay to these mixed DNA samples was recorded in a similar manner as mentioned in Section 2.6.

## 3. Results and Discussion

### 3.1. Optimization and Characterization of the Ink Formulation

**Figure 1:**
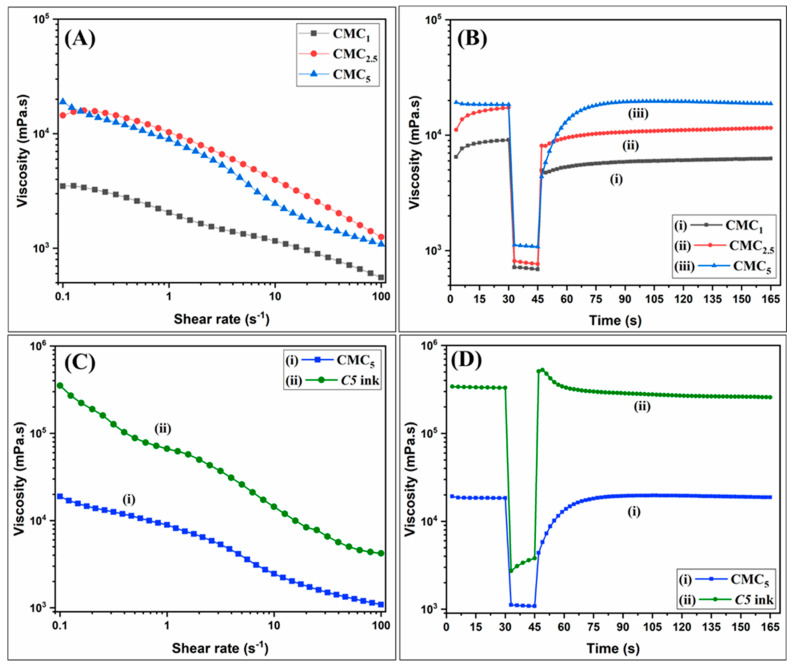
(**A**) Viscosity flow curves showing the change in viscosity of the CMC_1_, CMC_2.5,_ and CMC_5_ base emulsions with shear rate in the range of 0.1–100 s^−1^, (**B**) Change in viscosity of the CMC_1_, CMC_2.5,_ and CMC_5_ base emulsions with time when subject to sudden change in the shear rate, (**C**) Viscosity flow curves of the CMC_5_ base emulsion and the *C5* ink with shear rate in the range of 0.1–100 s^−1^, and (**D**) Change in viscosity of the CMC_5_ base emulsion and *C5* ink with time when subject to sudden change in shear rate.

The ink formulation was optimized and characterized for flow properties by rheometry. The aim of this study was to prepare a homogenous ink with thixotropic properties, which pertain to a linear decrease in a liquid’s viscosity with an increase in shear force followed by recovery of viscosity once the shear force is removed. Thixotropy allows for the smooth application of the ink on a particular substrate (in this case, paper) [29]. Most commercial paints and screen-printing inks are thixotropic in nature [29]. CMC is a known thixotropic agent and is prominently used as a rheological modifier [30]. To determine the effect of the amount of CMC on the ink rheology, three different solvent bases were prepared—CMC_1_, CMC_2.5,_ and CMC_5_—corresponding to 1%, 2.5%, and 5% CMC. Figure 1A shows the log plot of the change in viscosity with the shear rate in the range of 0.1–100 s^−1^. The viscosity varies linearly with the shear rate suggesting the shear thinning property of the three emulsion bases. The initial viscosities for CMC_1_, CMC_2.5,_ and CMC_5_ at 0.1 s^−1^ were found to be 3.48 Pa.s, 14.42 Pa.s, and 18.9 Pa.s, respectively. This increase in initial viscosity is due to the increase in CMC content in the solvent bases. The thixotropic behavior was further assessed by the 3-interval thixotropy test (3iTT) (Figure 1B). This test can simulate processes such as painting, screen printing, etc., by applying a sudden high shear stress to ink and then analyzing the percentage recovery in the viscosity of the ink after the stress is removed abruptly. CMC_5_ displayed the highest viscosity of 18.4 Pa.s at 0.1 s^−1^ (30 s), while CMC_1_ had the lowest viscosity (Figure 1B). In accordance with the shear thinning behavior, the viscosity of the emulsion bases decreased significantly at 200 s^−1^. After the removal of the stress, the viscosity of the three emulsions recovered to different degrees. For CMC_1_ and CMC_2.5_, the viscosities decreased to 0.68 Pa.s and 0.76 Pa.s at 45 s (10% and 4.78%, respectively, of their initial viscosities at 30 s). While for CMC_5_, the viscosity decreased to 1.08 Pa.s at 45 s (5.8% of initial viscosity at 30 s) indicating its higher viscosity in relation to the other two emulsion bases. This further suggests that CMC_1_ would perhaps flow the fastest and CMC_5_ would flow the slowest through the screen mesh while printing. After recovery of the shear rate from 200 s^−1^ to 0.1 s^−1^, the viscosity of CMC_1_ recovered to 62.5% of its original initial viscosity after 75 s. At the same time, CMC_2.5_ and CMC_5_ recovered 59.3% and 98.4% of their initial viscosities, respectively. Recovery of viscosity is crucial to determine the screen printability of inks as it indicates less seepage and dripping issues. For an efficient screen-printing ink, it is imperative that the ink should neither possess a very low viscosity (as it allows the creation of air bubbles) nor should it be too viscous, which leads to non-uniform printed patterns [29]. Since CMC_5_ displayed an appreciable decrease in viscosity and the highest recovery, it was chosen as the emulsion base for the formulation of the ink.

The *C5* ink was prepared by dispersing cMWCNT into the CMC_5_ emulsion base uniformly, and the effect of cMWCNT on the flow properties of the final ink was observed (Figure 1C,D). As shown in Figure 1C, the initial viscosity of the *C5* ink at 0.1 s^−1^ was found to be significantly higher (352.1 Pa.s) than the CMC_5_ emulsion. However, the ink retained its shear thinning property as the viscosity decreased linearly with the shear rate. It is also interesting to note that, during the thixotropy test (3iTT), the initial viscosity of the *C5* ink (329.8 Pa. s at 0.1 s^−1^ at 30 s) decreased by ~100 times to 3.79 Pa.s (~1% of the initial viscosity) at 200 s^−1^ at 45 s. The viscosity further recovered to 90% of its initial value at 75 s after the removal of the stress. This indicated that the addition of cMWCNTs did not affect the thixotropic behavior of the ink and hence the *C5* ink can potentially be used in screen printing of high-resolution patterns. Similar rheological behavior has been observed in other studies wherein MWCNT and other nanomaterials, such as silver, graphene, etc., were used as conductive fillers [29,31,32]. It has been reported that the presence of carbon nanomaterials as fillers in an ink increases the structural strength of the ink, and hence it may lead to a faster recovery of the viscosity [32]. The high viscosity recovery suggests that the colloidal stability of the ink is maintained even at higher shear rates, which results from the balance between the *van der Waals* interactions and steric stabilization among cMWCNTs, CMC (binder), and other dispersants (PS80) [31]. This eventually leads to better leveling during printing.

### 3.2. Morphological and Functional Characterization of C5@paper Electrodes

**Figure 2:**
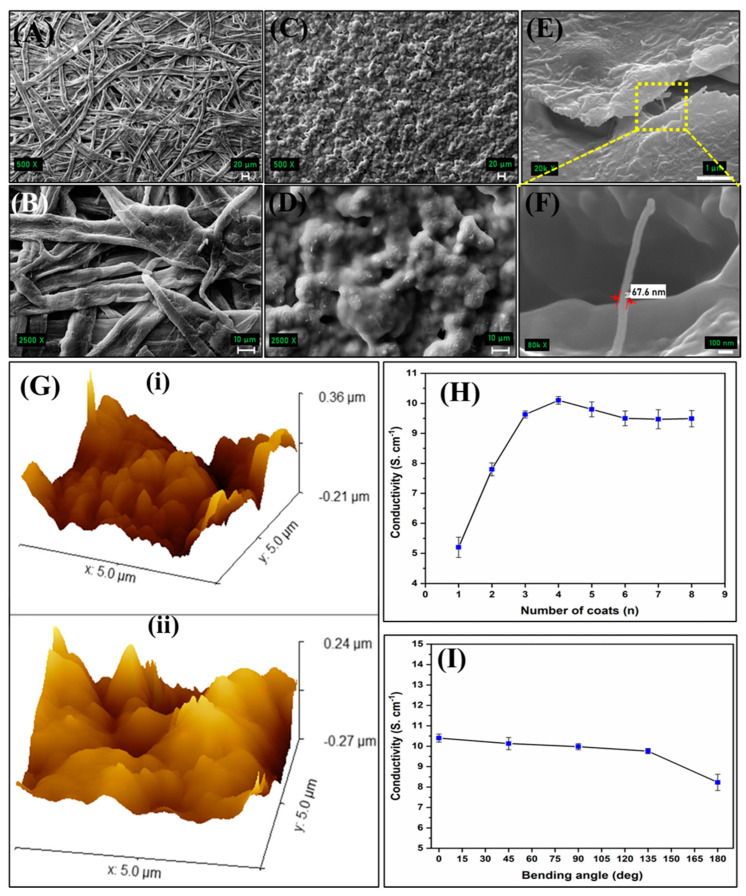
SEM images of (**A**,**B**) bare paper, and (**C**,**D**) *C5*@paper electrodes at different magnifications, (**E**) FESEM image of the *C5*@paper electrode, (**F**) High magnification FESEM image showing cMWCNT in the ink matrix, (**G**) AFM images of the (**i**) bare paper, and (**ii**) *C5*@paper electrode. Change in the conductivity of the *C5*@paper electrode with (**H**) number of coats, and (**I**) bending angle.

The morphology of the *C5*@paper electrodes has been studied by SEM and FESEM analysis (Figure 2A–F). The cellulose fibers of bare paper used as substrate are clearly visible in Figure 2A,B). These fibers are completely covered by the *C5* ink after coating, which appears uniform and has a granular appearance (Figure 2C,D). The cMWCNTs seem to be completely enveloped with CMC (and possibly PS80), though they are visible at some locations protruding out of the ink matrix (Figure 2D). Further, Figure 2E shows the magnified FESEM image of the tilted lateral side of the *C5*@paper electrode. The ink coats are clearly visible with cMWCNT dispersed over the entire surface. Upon further inspection of the lateral edge of the *C5*@paper electrode, it seems that the ink has not completely penetrated the entire thickness of the paper, as cellulose fibers are visible in between the ink coating (Figure S2). Figure 2F shows the magnified view of the *C5*@paper electrode with a cMWCNT protruding out of the ink matrix, having an apparent diameter of 67.6 nm. The surface roughness of bare paper and *C5*@paper electrodes was further analyzed by AFM studies (Figure 2G(i,ii), respectively). The RMS surface roughness of bare paper and the *C5*@paper electrode was found to be 168.2 nm and 41.6 nm, respectively. This reduction in the surface roughness upon coating may be contributed to the masking of cellulose fibers by the ink. This further suggests that, despite the grainy appearance, the ink coating is smooth and possesses lower friction force as compared to bare paper [33]. 

The conductivity of the *C5*@paper electrodes was measured by the 4-probe conductivity method. The calculations involved in the measurement of conductivity are given in Section S4. After coating, the electrodes were annealed at 120 °C for 10 min. The conductivity of the *C5*@paper electrodes was found to increase with the number of coats, with maximum value at four coats (*n* = 4), as shown in Figure 2H. This increase in conductivity can be attributed to the increase in the cMWCNT content after each coat. Interestingly, the conductivity was found to first reduce and then seemed to saturate gradually for 5–8 coats. The CMC concentration (% *w*/*v*) is equivalent to that of cMWCNT in the *C5* ink. Perhaps, the insulating nature of CMC starts dominating the conductivity after four coats and beyond. The saturation of the conductivity points toward the presence of competing factors contributed by the presence of conductive cMWCNTs and non-conductive binders (CMC) and, to some extent, emulsifiers (PS80). This is in contrast with a recent study that reported an increase in conductivity until saturation of a graphene-based ink with an increase in the number of (printing) coats [34].

The maximum conductivity of the *C5*@paper electrode was found to be 10.1 ± 0.24 S·cm^−1^ (0.1 ± 0.026 Ω·cm) after four coats of ink. A recent study has reported the conductivity of an MWCNT/PEDOT:PSS screen-printing ink to be 14.99 S·cm^−1^ for five printing passes [31]. It may be noted that the binder used in this study, PEDOT:PSS, is a conductive polymer, which played a significant role in the enhanced conductivity of the MWCNT ink. Another study reported the resistivity of an MWCNT ink in the range of 0.5–13 Ω·sq^−1^ [35]. The study reported the use of PVP as a binder and 10% *w*/*v* MWCNT as a conductive filler. The results of these studies highlight the importance of the binder used in ink preparation. The use of a conductive binder may enhance the conductivity manifold, while an insulating polymer may compromise the conductivity of ink even if the conductive filler is used in excess. Keeping this in view, the high conductivity of the *C5* ink, despite the use of a relatively smaller amount of MWCNT (5% *w*/*v*) and an insulating binder (CMC), alludes to the effectiveness of the method of ink preparation. Further, the change in conductivity of the *C5*@paper electrodes upon folding at different angles (45–180°) was also estimated (Figure 2I). There was no significant change in the conductivity of the electrodes until 135° bending. However, the conductivity reduced drastically upon a 180° bend. The retention of conductivity until 135° suggests that cMWCNTs are well dispersed within the ink matrix [36]. However, the surface of the paper may crack at higher bending angles (180°) leading to cracks in the ink itself. This ultimately contributed to lower conductivity values at higher bend angles. 

### 3.3. Electrochemical Studies

**Figure 3:**
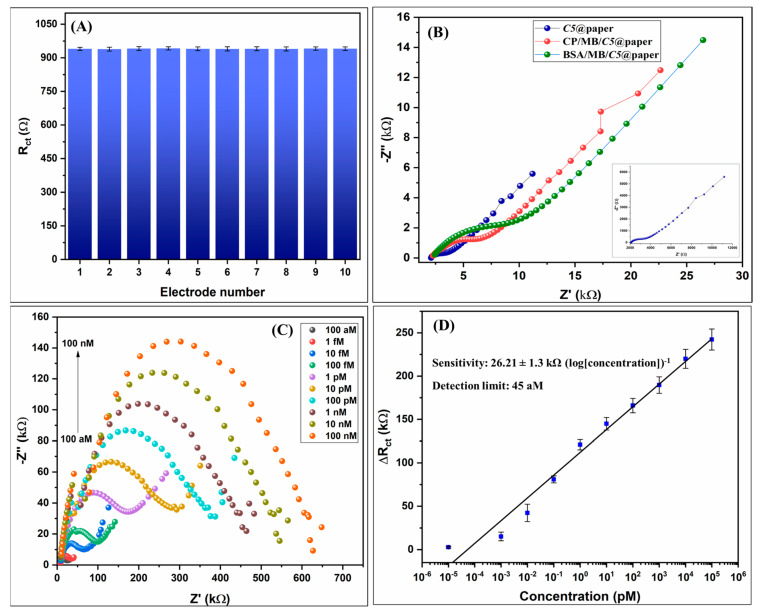
(**A**) Bar graph showing the R_ct_ values of C5@paper electrodes selected randomly from different batches. (**B**) Nyquist plots of the *C5*@paper, SCP/MB/*C5*@paper, and the BSA/SCP/MB/*C5*@paper electrodes. Inset shows the Nyquist plot of the *C5*@paper electrode. (**C**) Nyquist plots of the *C5*@paper after drop-casting of the SD-T-SC/MB for different concentrations of the TP. (**D**) Corresponding linear calibration curve showing ΔR_ct_ vs. log of the concentration of the TP.

The electrochemical performance of the fabricated *C5*@paper electrodes and further modifications were characterized via CV and EIS. Multiple CV cycles were run to determine the stability of the fabricated electrodes upon continuous use in the buffer. As shown in Figure S2, the response of the electrode does not change significantly even after 50 cycles, indicating the stability of the electrodes in buffer conditions. It may be noted that the charging current was found to be too high in relation to the faradaic current in the CV curves, which could be due to the pseudocapacitance of the electrode. The swelling of CMC in aqueous conditions may also contribute to the high charging current. For this reason, EIS was chosen for further electrochemical studies. The batch reproducibility of the *C5*@paper electrodes was also assessed by randomly selecting 10 electrodes from different batches and measuring their charge transfer resistance (R_ct_) values via EIS (Figure 3A). The R_ct_ values were determined by fitting the Nyquist plots by an equivalent model circuit given by (R_s_(Q_1_[R_ct_Q_2_])), wherein R_s_ is the solution resistance, Q_1_ is the constant phase element (CPE) associated with the (pseudo)capacitance of the electric double layer formed at the electrode surface, R_ct_ is the charge transfer resistance, and Q_2_ is the CPE associated with the diffusion of redox species at the electrode surface (Figure S3A). The presence of Q_1_ and Q_2_ in the model circuit indicates the non-ideal behavior of the double-layer capacitance. This also indicates that the distribution of active sites over the electrode surface might be non-homogenous [37]. As shown in Figure 3A, no significant difference was found in the R_ct_ of the *C5*@paper electrodes, with the mean R_ct_ being 940.29 ± 0.72 Ω. These results suggest that the cMWCNTs were dispersed homogenously in the *C5* ink, which led to the uniform coating of the paper substrates. The change in the R_ct_ of the *C5*@paper electrodes with time (1–30 days) was also studied to determine the storage life of the electrodes (Figure S3B). It was found that the initial R_ct_ at Day 1 (940 Ω) reduced by a mere ~0.5% on Day 30 (935.2 Ω), suggesting that the electrodes can be stored for at least 30 days at room temperature in a dry environment.

Figure 3B shows the Nyquist plots of the *C5*@paper electrodes and subsequent modifications. It can be seen that the R_ct_ (which is generally correlated to the diameter of the semicircle in the Nyquist plot) increases after every modification. The R_ct_ of the *C5*@paper was found to be 940 ± 4.2 Ω, which further increased to 3.28 ± 0.06 kΩ and 4.11 ± 0.09 kΩ, respectively, after modifications by SCP/MB and BSA/SCP/MB. This increase in R_ct_ indirectly indicates the immobilization of SCP and BSA onto the MB surface. The equivalent model circuit (R_s_(Q_1_[R_ct_Q_2_])) fitted into the plots is given in Figure S3A. Prior to the electrochemical response studies, gel electrophoresis studies were conducted to confirm the formation of the supersandwich between the SCP, TP, and SDP probes. As shown in Figure S4, DNA bands at ~100 bp and higher are observed at both 25 °C and 55 °C. Additionally, the supersandwich structures formed at both temperatures after a mere 5 min. Thus, 55 °C for 10 min was chosen as the optimal temperature and time for the DNA supersandwich hybridization studies. The higher temperature was chosen in order to avoid any non-specific interactions with non-gonorrheal DNA, which is often present in clinical samples. The time chosen was 10 min as the immobilization of SCP onto the MBs can significantly reduce their mobility in the aqueous phase. Steric hindrance can also hamper the rate of DNA hybridization. To compensate for such losses, the optimum incubation time was chosen as a bit higher than necessary. 

The Nyquist plots of the *C5*@paper electrodes for different concentrations of the TP are given in Figure 3C. A significant rise in the impedance was observed with increasing concentration of the target DNA, as is evident by the increasing semicircle diameter. The change in the R_ct_ (ΔR_ct_) was calculated by applying the following equation:

ΔR_ct_ = R_m_ − R_0_ (1)

where R_m_ is the measured R_ct_ for a given concentration and R_0_ is the R_ct_ of the negative control (electrode with no TP). The ΔR_ct_ values correlated linearly to the log of concentration of the *porA* target sequence (TP) (Figure 3D). The corresponding linear regression equation is:

ΔR_ct_ = 26.21 × log[concentration] + 111.99, R^2^ = 0.98 (2)

The linear range of the assay was found to be 100 aM–100 nM (10^9^ orders of magnitude) with an exceptional sensitivity of 26.21 kΩ (log[concentration])^−1^ and a detection limit of 45 aM, as calculated by a previously reported method [37]. The enhanced sensitivity can be contributed to the formation of supersandwich structures at the surface of the MBs. As shown in Table 2, the biosensing assay proposed in this work possesses a better detection limit than most recent studies reported on the development of electrochemical DNA biosensors. Further, the value of the detection limit (45 aM) is equivalent to 10–22 copies µL^−1^ corresponding to the longest and the shortest supersandwich DNA duplex length of 150 bp and 69 bp, respectively (Figure S3). This detection limit range is superior to those reported by recent studies on visual detection of NG via a multiple cross displacement assay (20 copies/test) and loop-mediated isothermal amplification (LAMP) assay (50 copies/test) [3,6]. In these studies, gold nanoparticles were used as the colorimetric probe. Luo et al. reported a similar detection limit of 10 copies µL^−1^ for a Cas13a-based specific high-sensitivity enzymatic reporter unlocking (SHERLOCK) assay developed for NG *porA* and azithromycin resistance detection [38]. It may be noted that this assay was tested on only purified double-stranded DNA (dsDNA). This demonstrates the superiority of the proposed biosensing assay in terms of a relatively simpler detection strategy, the use of fewer reagents, and label-free electrochemical detection. PCR is currently the gold standard for the detection of NG in clinical samples. Comparison with commercial PCR assays in terms of detection limit indicates that there is a scope for improvement in the proposed supersandwich biosensing assay. GeneProof^®^ claims the detection limit of its NG dual-target PCR kit to be 0.109 copies µL^−1^ [39]. While another study has reported the detection limit of the same assay being 1.5 copies µL^−1^ [40]. It is important to note here that PCR is an amplification-based assay, and, hence, in some instances, the detection limit is simply defined as being the lowest concentration returning amplification. Using this approach, a study reported the assay detection limit as being 1 × 10^−7^ dilution [41], while another reported a detection limit of 10^2^–10^3^ copies mL^−1^ (0.1–1 copy µL^−1^) [42]. Thus, readers are advised to consider these factors while analyzing the differences between biosensing assays and PCR assays and their respective detection limits.

### 3.4. Real Sample Analysis and Specificity Studies

**Figure 4:**
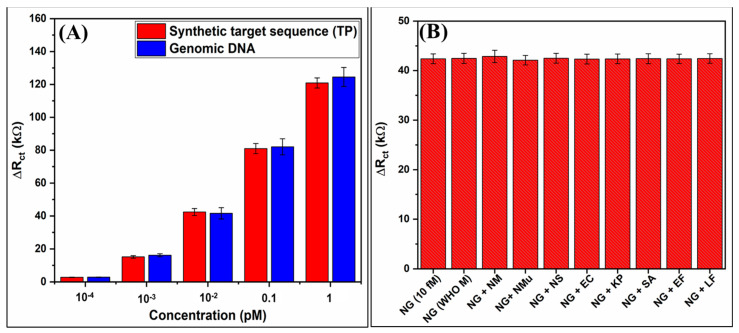
(**A**) Comparison between the biosensor response to synthetic TP and genomic DNA from NG for different concentrations (100 aM–1 pM). (**B**) Response of the biosensing assay to NG DNA mixed with DNA from other bacterial species. NM: *Neisseria meningitidis*, NMu: *Neisseria mucosa*, NS: *Neisseria sicca*, EC: *Escherichia coli*, KP: *Klebsiella pneumoniae*, SA: *Staphylococcus aureus*, EF: *Enterococcus faecalis*, LF: *Lactobacillus fermentum*.

To determine whether the electrochemical biosensing assay would perform in clinical conditions, its response was recorded against different concentrations of genomic DNA extracted from NG in the concentration range of 100 aM–1 pM. As shown in Figure 4A, there was no significant change in the ΔR_ct_ of the biosensing electrode for the same concentrations of the genomic DNA and synthetic TP used in the response studies. This indicates the applicability of the biosensing assay in real-life situations.

The specificity of the biosensing assay was assessed by mixing the genomic DNA of NG with DNA from other bacteria (Section S3). The DNA was extracted using the boiling method; hence, all samples may contain protein and RNA as contaminants. The NG genomic DNA was mixed with DNA from other bacteria and subjected to the protocol mentioned in Section 2.7. As shown in Figure 4B, no significant change in ΔR_ct_ of the *C5*@paper biosensing electrode was observed when recorded in the presence of interfering DNA from different bacterial species. The ΔR_ct_ values for the different DNA mixtures are given in Table S1. These studies highlight the significance of sample enrichment via MBs before biosensing and hint at the high specificity of the designed oligonucleotide probes. 

## 4. Conclusions

We have fabricated a conductive ink-coated, paper-based biosensing assay for the ultrasensitive detection of *Neisseria gonorrhoeae* using EIS. *C5*, the MWCNT-based conductive ink used in this study, was found to be highly conductive, with the maximum conductivity of the *C5*@paper electrodes being 10.1 S·cm^−1^. The ink formulation was optimized for thixotropy that can allow batch reproduction by screen printing. A magnetic bead-assisted supersandwich DNA hybridization assay was designed, which upon integration with the *C5*@paper electrodes, was found to be highly sensitive with an excellent detection limit (45 aM). The biosensing assay was further found to be highly specific for gonorrheal DNA when tested in a mixture of DNA from different non-gonorrheal species. This study not only reveals the utility of paper-based biosensors for DNA diagnostics but also highlights the potential of conductive inks in the fabrication of paper-based biosensors. It further attempts to bring gonorrhea to the forefront of scientific research as an important biosensing target. Further studies should focus on the modification of the conductive inks for obtaining improved electrochemical performance, which would further enhance biosensor sensitivity. 

## Figures and Tables

**Table 1:** Oligonucleotide probes used in the study.

S. No.	Probe Type	Probe Name	Sequence (5′-3′)	Melting Temperature (°C)
1.	Capture probe	SCP	5′ NH_2_-TTT TTT CCT GCT ACT TTC ACG CTG GA 3′ (26 bases) (5′ amino C6 modification)	59
2.	Detector probe (supersandwich)	SDP	5′ AAG TAA TCA GAT GAA ACC AGT TCC CTG CTA CTT TCA CGC TGG A 3′ (43 bases)	50.8
3.	Target strand	TP	5′ GAA CTG GTT TCA TCT GAT TAC TTT CCA GCG TGA AAG TAG CAG G 3′ (43 bases)	66.2
4.	Non-complementary probe	NC	5′ TTG ATT GAG CGT CAT AGA TCG GAC GGC ACT AGT CAG TAC TCG A 3′ (43 bases)	67

**Table 2:** Comparison of recent studies on paper-based biosensors for DNA detection.

S. No.	Ink Formulation/ Nanomaterial	Type of DNA Assay	Target Pathogen	Linear Range	Sensitivity (LOD)	Reference
1.	Ni–Au composite/CNT/PVA	Traditional hybridization	HIV DNA	10 nM–1 µM	(0.13 nM)	[26]
2.	Tungsten disulfide quantum dots	Traditional hybridization	Specific meningitis	1 nM–100 µM	(1 nM)	[43]
3.	Oxidized graphitic carbon nitride	Traditional hybridization	Norovirus	10^−7^–10^2^ µM	(100 fM)	[25]
3.	Graphene nanosheets	Direct analysis	(dsDNA)	0.2–5 pg mL^−1^	0.00656 mA·pg^−1^ cm^−2^	[24]
4.	Carbon graphene ink (commercial)	Traditional hybridization (pyrrolidinyl peptide nucleic acid-mediated)	Hepatitis B virus	50 pM-100 nM	(1.45 pM)	[44]
5.	Ag nanoprisms electrodeposited on graphene quantum dots (GQDs)	Traditional hybridization	*Leishmania infantum*	1 zM–1 nM	1 zM (LOQ *)	[45]
6.	cMWCNT	Supersandwich hybridization	*Neisseria gonorrhoeae*	100 aM-100 nM (5 zmol-5 pmol)	26.21 kΩ (log[concentration])^−1^ (45 aM)	This work

## Data Availability

Data is contained within the articles and the supplementary information.

## Supplementary Materials

The following supporting information can be downloaded at https://www.mdpi.com/article/10.3390/bios13040486/s1. Section S1: Alignment of the designed oligonucleotide probes with the target sequence (porA pseudogene); Section S2: Protocol followed for SCP immobilization on the MBs; Section S3: Bacterial species used in the specificity studies; Section S4: Calculation of resistivity of the *C5*@paper electrodes; Figure S1: FESEM image of the lateral edge of *C5*@paper electrode; Figure S2: CV curves of the *C5*@paper electrodes showing continuous use stability; Figure S3A: Equivalent circuit fitted into the Nyquist plots; Figure S3B: Bar graph showing the R_ct_ of *C5*@paper electrodes during 1-30 days of storage; Figure S4: Gel electrophoresis studies to ascertain the formation of DNA supersandwich structures; Table S1: R_ct_ values corresponding to 10 fM NG DNA mixed with DNA from different bacterial species.
