# Supplementary material for: Conductive Ink-Coated Paper-Based Supersandwich DNA Biosensor for Ultrasensitive Detection of Neisseria gonorrhoeae
Source: Biosensors (Basel). 2023 Apr 18;13(4):486. doi: 10.3390/bios13040486 (PMC10136323; doi:10.3390/bios13040486)
Supplement: Supplementary file 1 [file biosensors-13-00486-s001.zip › biosensors-2284275-supplementary.docx]

**Supplementary Information**

**Conductive ink-coated paper based supersandwich DNA biosensor for ultrasensitive detection of *Neisseria gonorrhoeae***

Niharika Gupta^1^, D. Kumar^2^, Asmita Das^1^, Seema Sood^3^, Bansi D. Malhotra^1,*^

^1^Department of Biotechnology, Delhi Technological University, New Delhi 110042 INDIA

^2^Department of Applied Chemistry, Delhi Technological University, New Delhi 110042 INDIA

^3^Department of Microbiology, All India Institute of Medical Sciences, Ansari Nagar, New Delhi 110016 INDIA

Corresponding Author: [bansi.malhotra@gmail.com](mailto:bansi.malhotra@gmail.com)

**S1. Alignment of the designed oligonucleotide probes with the target sequence (porA pseudogene)**

5’NH_2_- TTT TTT CCT GCT ACT TTC ACG CTG GA 3’ Capture (SCP)

3’GGA CGA TGA AAG TGC GAC CT TTC ATT AGT CTA CTT TGG TCA AG 5’ Target (TP)

5’AAG TAA TCA GAT GAA ACC AGT TCC CTG CTA CTT TCA CGC TGG A 3’ Detector (SDP)

3’ G GAC GAT GAA AGT GCG ACC T TTC ATT AGT CTA CTT TGG TCA AG 5’ (TP)

5’AAG TAA TCA GAT GAA ACC AGT TCC CTG CTA CTT TCA CGC TGG A 3’ SDP

…… (TP)…… so on

5’NH_2_- TTT TTT CCT GCT ACT TTC ACG CTG GA 3’ SCP

3’GGA CGA TGA AAG TGC GAC CT TTC ATT AGT CTA CTT TGG TCA AG 5’ TP

5’AAG TAA TCA GAT GAA ACC AGT TC 3’ Detector (DP)

**S2. Protocol followed for SCP immobilization on the MBs**

*SCP immobilization.* SCP was immobilized onto the carboxylated MBs using carbodiimide chemistry and following the manufacturer’s protocol with slight modifications. Briefly, the carboxylated MBs were washed with 0.01 M NaOH (1 ×; 10 min) and DI water (3×). Freshly prepared EDC (50 mg mL^-1^) and NHS (150 mg mL^‑1^) were added to these MBs, which were then incubated with mixing at room temperature for 45 min. Thereafter, the thus modified MBs were washed with DI water (5×) to remove extra EDC/NHS and were incubated with SCP (2000 pmol per mg MBs) with mixing at room temperature for 1 h. The SCP-modified MBs (SCP/MB) were washed with DI water (5 ×) to remove non-specifically bound and unbound SCP. This was followed by blocking with 0.1% BSA and PBS (pH 8). The thus prepared SCP/MB were stored in TE buffer with a final concentration of 10 mg mL^-1^ at 4 °C till further use.

**S3. Bacterial species used in the specificity studies**

*Neisseria meningitidis*, NM; *Neisseria mucosa*, NMu; *Neisseria sicca*, NS; *Escherichia coli*, EC; *Klebsiella pneumoniae*, KP; *Staphylococcus aureus*, SA; *Enterococcus faecalis*, EF; *Lactobacillus fermentum*, LF

**
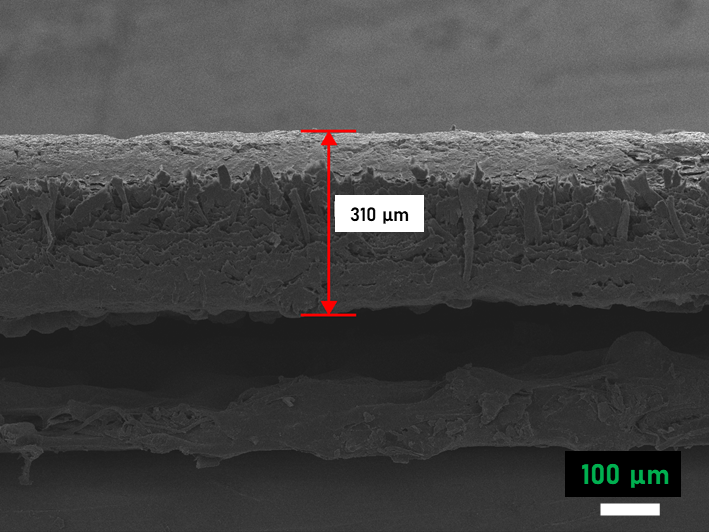
**

**Cellulose fibers**

**Figure S1.** FESEM image of the lateral edge of C5@paper electrode showing the cellulose fibers beneath the C5 ink coating. The total thickness of the C5@paper electrode is 310 µm.

**S4. Calculation of resistivity of the *C5*@paper electrodes**

The following equations were utilized in determining the resistivity of the cMWCNT@paper electrodes:

ρ =$\frac{\rho o}{G'}$ (Eq. 1)

where, ρ is the final resistivity corrected for the thickness of coating, ρ_o_ is the resistivity given by the following equation:

ρ_o_ = $\left( \frac{V}{I}\times2\pi S \right)$ (Eq. 2)

where, V is the potential, I is the current and S is the distance between the probes of the conductivity meter (0.2 cm), and G′ is the error introduced when the thickness of the conductive layer, t << S and is given by:

G′ = $\frac{2S}{t}\ln2$ (Eq. 3)

The conductivity of the sample, σ is given by,

σ = 1/ ρ (Eq. 4)





**Figure S2.** CV curves of the C5@paper electrode at first cycle, 25^th^ cycle and 50^th^ cycle displaying the continuous use stability. Inset shows a magnified view of the CV curves.


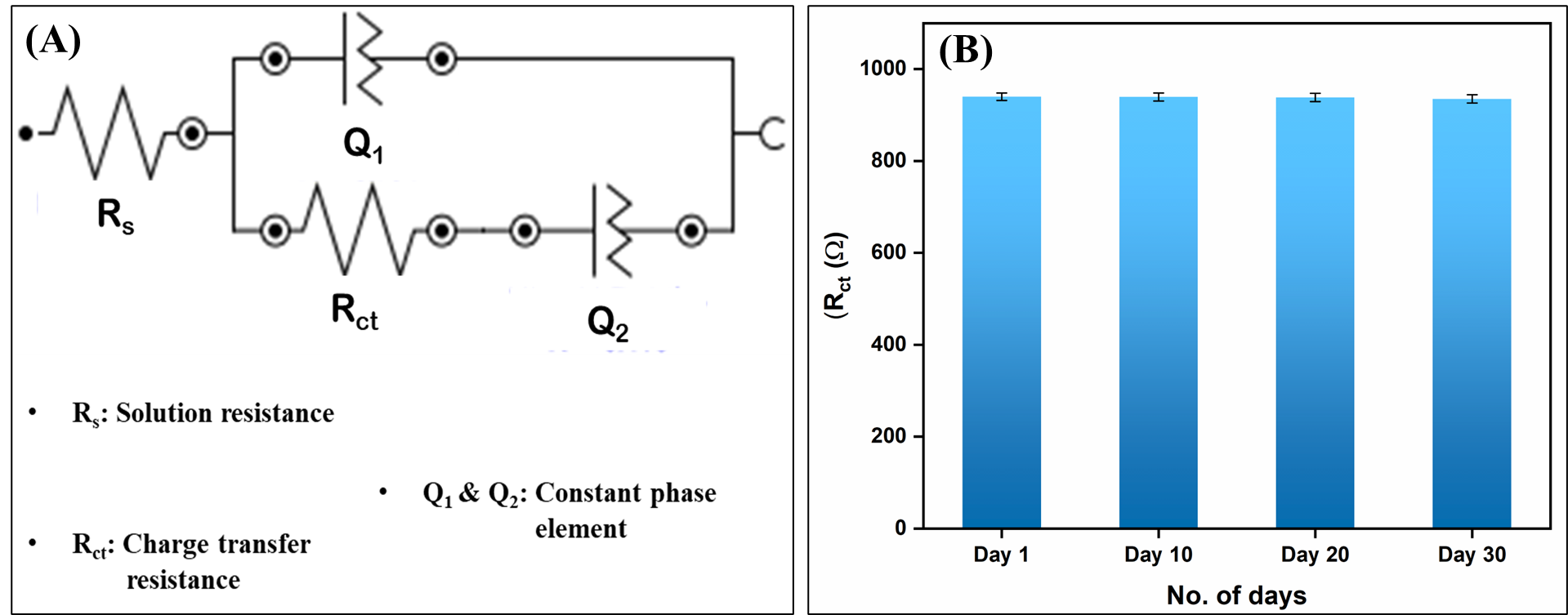


**Figure S3.** **(A)** Equivalent circuit model fitted into the Nyquist plots. **(B)** Bar graph showing the R_ct_ of C5@paper electrode during 1-30 days of storage in dark, dry conditions at room temperature.


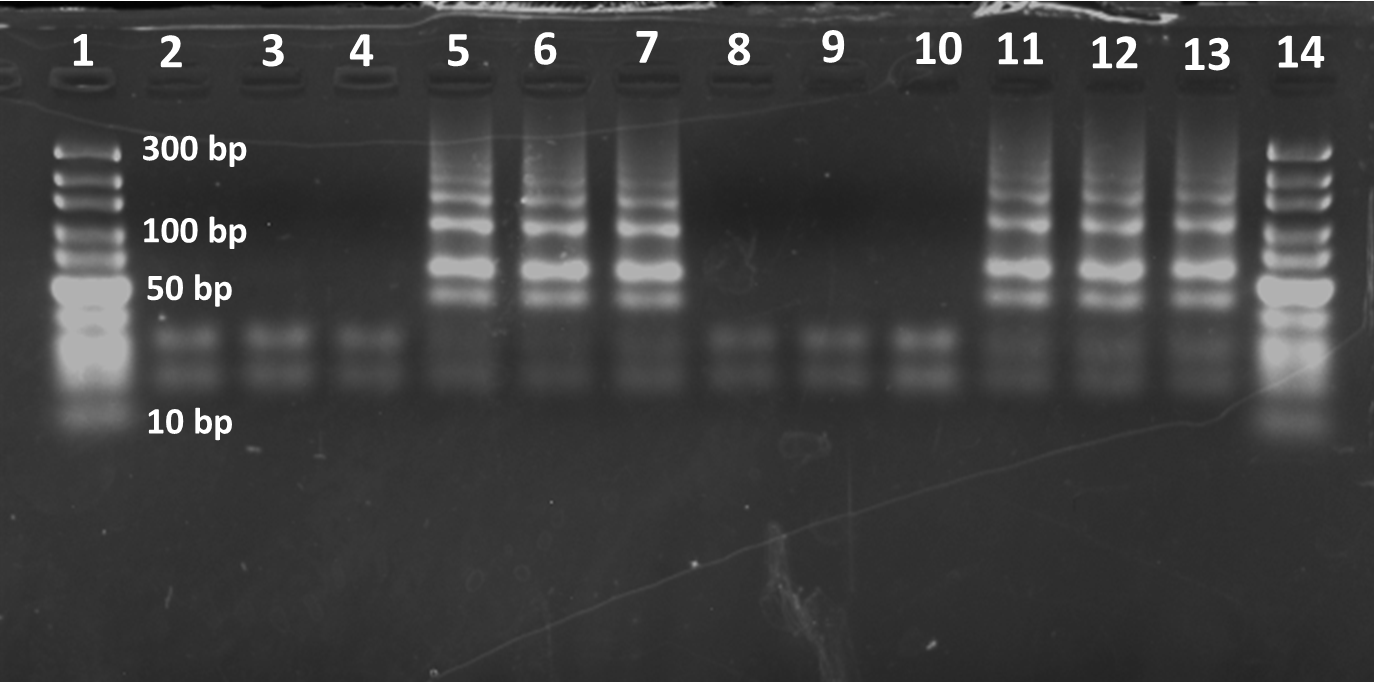


**Figure S4.** Gel electrophoresis studies to ascertain the formation of DNA supersandwich structures and optimizing the temperature and time of incubation. Lane 1, 14: DNA ladder (10-300 bp), Lanes 2-4: SCP and SDP without TP incubated at 25 °C for 5, 10 and 15 min respectively, Lanes 5-7: Supersandwich structures formed by hybridization of SCP, TP and SDP at 25 °C for 5, 10 and 15 min, respectively, Lanes 8-10: SCP and SDP without TP incubated at 55 °C for 5, 10 and 15 min respectively, and Lanes 11-13: Supersandwich structures formed by hybridization of SCP, TP and SDP at 55 °C for 5, 10 and 15 min, respectively.

**Table S1. The ΔR_ct_ values of 10 fM NG DNA mixed with DNA from different bacterial species**

| **S. No.** | **DNA from bacteria** | **ΔR_ct_ (mean, kΩ)** |
| --- | --- | --- |
| 1. | NG (Synthetic TP, 10 fM) | 42.39 |
| 2 | NG (WHO M, 10 fM) | 42.46 |
| 3 | NG + N. meningitidis (NM) | 42.87 |
| 4 | NG + N. mucosa (NMu) | 42.1 |
| 5 | NG + N. sicca (NS) | 42.5 |
| 6 | NG + E. coli (EC) | 42.33 |
| 7 | NG + K. pneumoniae (KP) | 42.37 |
| 8 | NG + S. aureus (SA) | 42.4 |
| 9 | NG + Enterococcus faecalis (EF) | 42.38 |
| 10 | NG + Lactobacillus fermentum (LF) | 42.44 |
